# Lysophosphatidic Acid and Ion Channels as Molecular Mediators of Pain

**DOI:** 10.3389/fnmol.2018.00462

**Published:** 2018-12-12

**Authors:** Rebeca Juárez-Contreras, Tamara Rosenbaum, Sara L. Morales-Lázaro

**Affiliations:** División de Neurociencias, Instituto de Fisiología Celular, Departamento de Neurociencia Cognitiva, Universidad Nacional Autónoma de México, Ciudad de México, Mexico

**Keywords:** LPA, ion channel, pain, TRPV1, Nav1.8, K2P, Cav3.2

## Abstract

Lysophosphatidic acid or LPA is a phospholipid which has been extensively linked to the generation and maintenance of pain. Several ion channels have also been shown to participate in this pathological process but the link between LPA and these proteins in pain has just recently gained interest. In this respect, the field has advanced by determining the molecular mechanisms by which LPA promotes changes in the function of some ion channels. While some of the actions of LPA include modulation of signaling pathways associated to its specific receptors, other include a direct interaction with a region in the structure of ion channels to affect their gating properties. Here, we focus on the known effects of LPA on some transient receptor potential, sodium, potassium, and calcium channels. As the field moves forward, mechanisms are unveiled with the hope of understanding the underlying causes of pain in order to target these and control this pathophysiological state.

## Introduction

Nociceptors are peripheral sensory neurons that respond to a wide diversity of harmful stimuli and transduce these stimuli into signals that reach the brain (Dubin and Patapoutian, 2010), leading to the subjective experience of pain defined as an “unpleased sensory and emotional experience associated with actual or potential tissue damage” (*International Association of Pain Study*).

**Figure 1:**
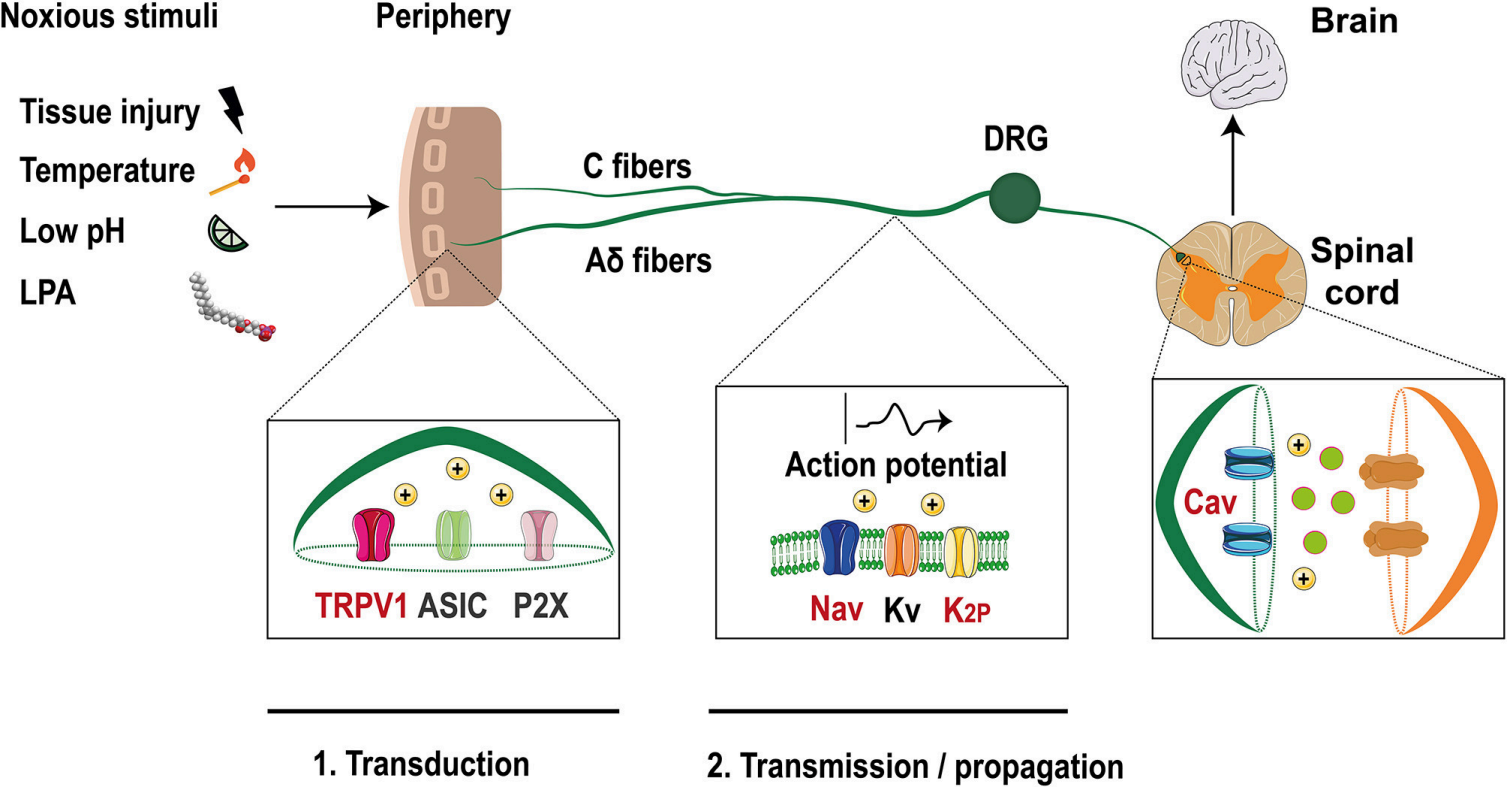
Ion channels in the pain pathway modulate by LPA. Noxious stimuli that include LPA, are transduced through nociceptors in the free termini (C and Aδ fibers) to dorsal horn neurons localized in the spinal cord. Finally, the signals travel to the brain where they are decodified. Ion channels such as TRPV1, ASIC, and P2X are activated by these stimuli producing membrane depolarization. Then, voltage-gated ion channels (Nav, Kv) and two pore domain potassium channels (K_2_P) are activated and the action potential is propagated along the axon. Finally, membrane depolarization activates voltage-gated calcium channels (Cav) leading to the release of excitatory molecules at the synapses. Ion channels modulated by LPA are shown with red letters (Modified from Waxman and Zamponi, 2014).

Neuronal activity is initiated at these specialized neurons, with somas located at the dorsal root ganglia (DRG) or trigeminal ganglia (TG) (Dubin and Patapoutian, 2010). The axons of these neurons split into two branches with one innervating peripheral organs and the other projecting to the dorsal horn or to the brainstem (Figure 1). These nociceptors are classified into Aδ fibers which are medium-size and lightly myelinated fibers with a conduction velocity of ~5–30 m/s or as small diameter unmyelinated C fibers that have lower conduction velocity (~0.4–1.4 m/s) (Dubin and Patapoutian, 2010).

Nociceptors contain a huge diversity of specialized membrane proteins such as G-protein-coupled receptors (GPCRs) that regulate neuronal physiology and ion channels that form highly regulated gates that control the influx of ions through the plasma membrane, resulting in changes of the membrane potential.

Noxious inputs trigger the activation of ion channels such as: acid sensing ion channels (ASIC), P2X purinoreceptors and members of the transient receptor potential (TRP) family, such as the TRPV1 (vanilloid) channel (Basbaum et al., 2009). Their activation leads to membrane depolarization initiating the first step of the pain pathway where noxious signals are converted into electrical messages (transduction) (McEntire et al., 2016).

If the magnitude of depolarization is sufficient, voltage-gated sodium channels (Nav) are activated and an action potential (AP) is initiated and propagated along the axon (transmission) until it reaches the synaptic zone, where the activation of voltage-gated calcium channels (Cav) produces the release of excitatory neurotransmitters (Sekiguchi et al., 2018). Furthermore, voltage- gated potassium channels (Kv) are expressed in nociceptors and their activation leads to membrane repolarization and hyperpolarization (i.e., two pore domain potassium channels, K_2P_), inhibiting the signaling pathway, and allowing the neurons to respond again (Ocana et al., 2004).

An endogenous chemical mediator of pain produced during tissue injury or inflammation states is lysophosphatidic acid (LPA) (Eichholtz et al., 1993), a phospholipid that regulates the expression/function of ion channels in the pain pathway.

**Figure 2:**
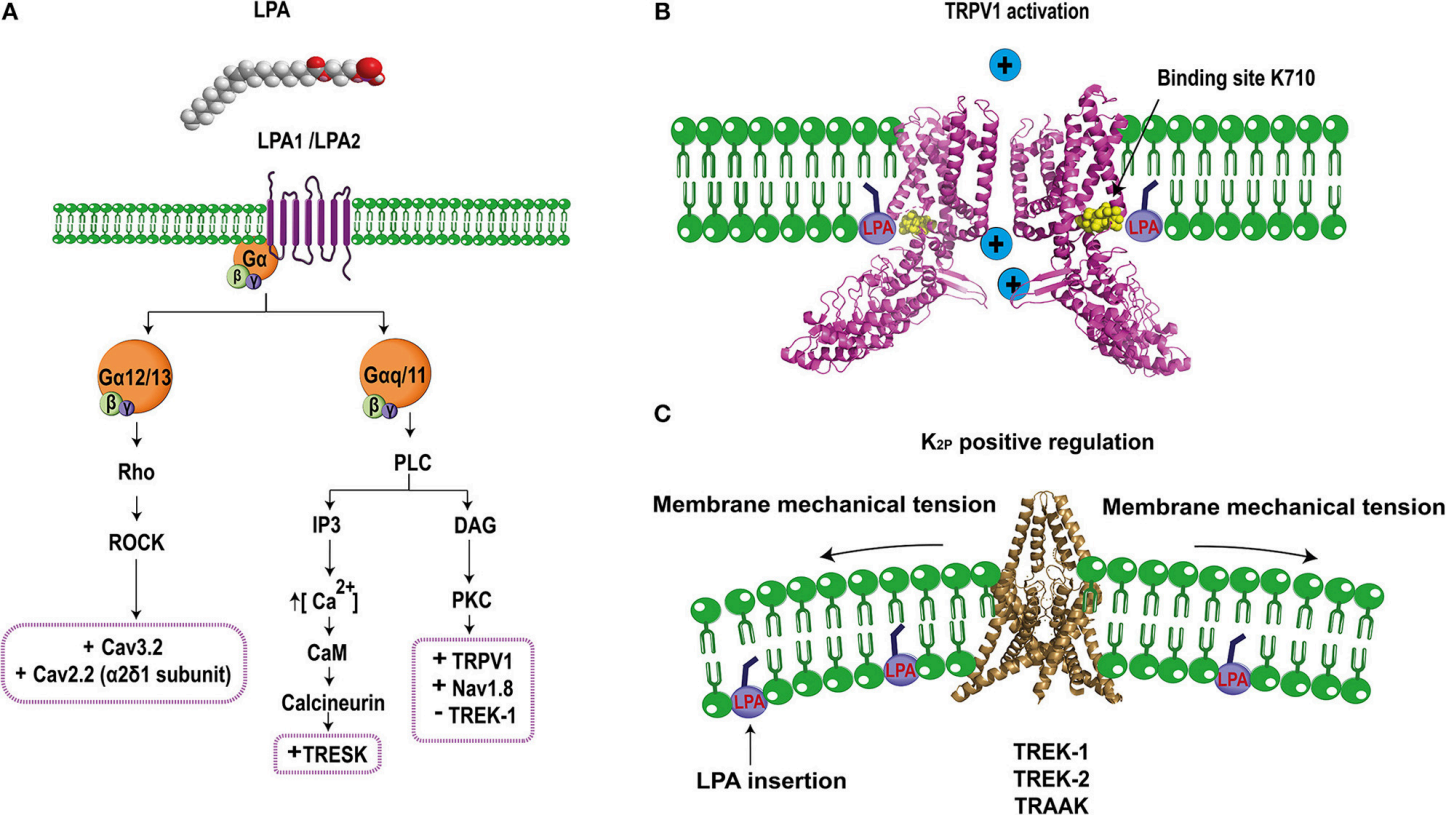
Schematic molecular mechanism of LPA's effects on ion channel regulation. **(A)** LPA (1- or 2-acyl-sn-glycero-3-phosphate) binds to LPA_1_ or LPA_2_ receptors regulating specific ion channels in the pain pathway. LPA_1/2_ receptor are coupled to the G_α12/13_ signaling pathway that activates the Rho cascade, positively regulating (+) Cav3.2 and the α2δ1 subunit of Cav2.2 channels. Furthermore, the LPA_1/2_ receptors are also coupled to G_α*q*/11_ leading to PLC (phospholipase C) activation to produce IP3 (inositol-3 phosphate) and DAG (diacylglycerol). IP3 increases the release of intracellular calcium which activates calcium-dependent enzymes such as CaM (calmodulin) and calcineurin and the latter positively regulates (+) TRESK channels. In addition, DAG can also activate PKC (protein kinase C) which positively regulates (+) TRPV1 and Nav1.8 but negatively (–) regulates TREK1 channels. **(B)** Direct interaction of LPA through binding with lysine 710 located in the C-terminus of TRPV1 produces channel activation allowing for ion influx (PDB 3J5R Liao et al., 2013). **(C)** TREK channels are positively regulated by LPA, this effect is probably produced by the insertion of the phospholipid into the plasma membrane, causing a change in the membrane curvature that modifies the activity of these mechanosensitive channels (PDB 4TWK; Miller and Long, 2012).

LPA is composed of a glycerol backbone with an ester link to a phosphate head group and an acyl fatty tail (Figure 2). This tail is usually constituted by 16 to 20 carbons linked by single (saturated) or double (unsaturated) bonds giving rise to different LPA species (Gerrard and Robinson, 1989). LPA is mainly produced through autotaxin, an ectoenzyme that uses lysophospholipids as precursor molecules (Tokumura et al., 2002) or by enzymatic actions of phospholipases A1 or A2 (PLA1/2) using phosphatidic acid as substrate (Aoki et al., 2002).

LPA's biological effects are broad and include participation in cell growth, differentiation, proliferation, survival, motility, cytoskeleton changes, and pain (Yung et al., 2014). Classical LPA actions are mediated by six specific GPCRs: LPA_1−6_ (Yung et al., 2014). These trigger different signaling pathways through Gα proteins (G_i_, G_q_, G_12_, and G_s_) producing different tissue responses such as in the nervous system, where LPA signaling has an important role in neuronal development and myelination (Yung et al., 2015). Importantly, activation of these receptors also leads to the generation of pain in fully-developed model animals (Inoue et al., 2004).

This review focuses on the effects of LPA on some ion channels expressed in sensory neurons and discusses the relevance of the regulation of these proteins by LPA in pain. In particular, we discuss the effects of LPA on TRPV1, Nav1.8, TRESK, TREK1, Cav3.2 channels, and the α2δ1 subunit of Cav2.2 channels, which are the only channels shown to be regulated by LPA to date.

## Activation of TRPV1 Channels by LPA

Several noxious signals are converted into electrical ones through TRP ion channels. The better studied member of this family is TRPV1, which is widely expressed in small diameter neurons of the DRG and TG (Caterina et al., 1997) and is activated by temperatures ≥43°C, extracellular acid and intracellular basic pHs, pungent chemical compounds, as capsaicin, resineferatoxin, allicin, and by some toxins (Szolcsanyi and Sandor, 2012). Moreover, some lipidic compounds produced during inflammation or tissue injury (i.e., anandamide, metabolites of lipoxygenase, and LPA) function as endogenous activators of TRPV1 producing pain (Morales-Lazaro et al., 2013).

The first connection between LPA and TRPV1 was described in rat DRG neurons, where the co-expression of this channel with the LPA_1_ receptor (associated to pain), was determined. Electrophysiological experiments in DRGs showed a dose-dependent potentiation of capsaicin evoked currents by LPA, which were blocked by inhibitors of the LPA_1_ receptor and PKCε (Figure 2A), demonstrating that downstream activation of PKCε by the LPA_1_ receptor pathway is crucial for TRPV1 sensitization (Pan et al., 2010).

Shortly after this, our group demonstrated the direct activation of TRPV1 by LPA and showed that this phospholipid produces acute pain through this channel when it is injected into the paws of mice (Nieto-Posadas et al., 2011). This pain-like behavior produced by LPA was decreased in the TRPV1 knockout (KO) mice, strongly indicating that LPA requires TRPV1 to transduce this noxious input. In addition, by using a mouse model where the gene that encodes for the enzyme that dephosphorylates LPA (LPP3) was conditionally deleted and where LPA levels are augmented in the nervous system, we showed that these animals exhibited thermal hyperalgesia and increased pain, as compared to the wild-type (WT) mice (Nieto-Posadas et al., 2011).

Furthermore, we also demonstrated that LPA elicited action potential firing in WT mouse DRGs, whereas this electrical activity was not observed in DRG neurons from TRPV1 KO mice. We detailed TRPV1 activation by LPA in excised membranes patches from TRPV1-expressing HEK293 cells and found that TRPV1 currents were generated upon intracellular and extracellular LPA application (Nieto-Posadas et al., 2011) and that this phospholipid produced a different conformational change to that produced by capsaicin, allowing for a larger conductance state (Canul-Sanchez et al., 2018).

We next assessed the role of LPA receptors on the activation of TRPV1 and showed that these where not responsible for activation of TRPV1. These data led us to propose and to demonstrate a direct interaction between LPA and TRPV1 (Figure 2B). By combining mutagenesis, *in vitro* interaction assays and electrophysiology, we showed that LPA binds to a lysine located on the carboxy- end of TRPV1 (K710), (Nieto-Posadas et al., 2011), a binding site shared with PIP2 (an anionic lipid that regulates the function of TRPV1) (Ufret-Vincenty et al., 2011). Altogether, these results demonstrated that LPA produced acute pain through a direct activation of TRPV1 channel.

The structural determinants of LPA required for activation of TRPV1 are: a monounsaturated long acyl chain (18 carbons for natural phospholipids and from 18 to 20 carbons for the synthetic analogs) and an anionic head-group (Morales-Lazaro et al., 2014).

Another TRP channel that is activated by LPA is TRPA1 in which the phospholipid directly interacts with positively-charged residues located in the amino-end (K672-K673) and in the carboxy-end (K977-R978) of the channel (Kittaka et al., 2017). However, that study reported that the physiological response to LPA injection in mice is mostly an itch behavior and not one of pain.

## LPA as a Regulator of Sodium Currents on Sensory Neurons

As mentioned before, the action potential is initiated and propagated along the axons of primary neurons due to the electrical activity of Nav channels. These are formed by α and β subunits with nine independent genes coding for the α subunits that produce the Nav1.1-1.9 channels, among which Nav1.7-1.9 have been associated to pain (Theile and Cummins, 2011).

A relationship between LPA-dependent pain and these channels was determined in rat small DRG neurons, where sodium currents were shown to exhibit increased tetrodotoxin (TTX)-resistant currents in the presence of LPA (Seung Lee et al., 2005). These neurons express TTX-resistant Nav1.8 and Nav1.9 channels (Ho and O'Leary, 2011); thus, the data suggested that LPA positively regulates these currents essential for the pain pathway.

Experiments also showed that intrathecal injection of LPA upregulates the expression of the Nav1.8 channel together with the LPA_1_ receptor in rat DRG neurons, effects that were blocked when LPA was co-injected with an antagonist of the LPA_1_ receptor (Pan et al., 2016). Additional experiments performed on small DRG neurons, where the membrane potential was maintained at −60 V (to inhibit currents produced by Nav1.9 channels) and LPA was perfused, showed Nav1.8-current potentiation indicating that the activity of this specific channel is positively regulated by LPA (Pan et al., 2016).

LPA effects on sodium currents were also reported in a rat bone cancer model, where the animals exhibit severe hyperalgesia, displaying LPA_1_ receptor upregulation and showing a higher percentage of LPA-sensitive C-fibers than in control animals (Pan et al., 2016). In this model, the expression of Nav1.8 channels is upregulated and these are widely co-localized with LPA_1_ in DRG neurons. Moreover, animals treated with an inhibitor of the LPA_1_ receptor, exhibited a decrease in the expression of Nav1.8 channels and partial attenuation of mechanical hyperalgesia (Pan et al., 2016).

Evidences obtained with this model and through intrathecal LPA injection demonstrated a crosstalk between Nav1.8 channels and the LPA_1_ receptor, suggesting the participation of a downstream pathway (Figure 2A). Further experiments using an inhibitor of PKCε showed that Nav1.8 current potentiation are mediated through this intracellular signaling pathway (Pan et al., 2016).

## Two Pore Domain Potassium Channels (K_2P_) are Targets of LPA

The resting membrane potential of cells is controlled by potassium channels, since these facilitate repolarization and hyperpolarization of the membrane and regulate pain (Du and Gamper, 2013). Some members of the potassium channel family are expressed in nociceptors: K_V_, calcium activated potassium (K_Ca_) and two pore domain potassium (K_2P_) channels (Du and Gamper, 2013). Although, some reports have shown that LPA regulates their function, trafficking or expression, few studies have shown their relationship to the pain pathway.

For example, it has been reported that some K_2P_ channels are targets of LPA, contributing to the modulation of pain. TRESK, TREK1-2, TRAAK, TALK1, TASK1-2 channels are expressed in DRG and the TRESK channel is the most abundant K_2P_ in DRG neurons (Dobler et al., 2007). The latter importantly contributes to the background K^+^ conductance in nociceptors and plays a role in the excitability of these neurons. TRESK's role in nociception has been demonstrated in DRG neurons from mice lacking its expression where less current input is needed to elicit action potentials (Cohen et al., 2009) and in TG neurons that over-express this channel and display reduced excitability as well as a significant reduction in the spike frequency in response to capsaicin (Guo and Cao, 2014). The TRESK channel is co-expressed with the LPA_2_ receptor and TRPV1 ion channels in small diameter mice DRG neurons (Kollert et al., 2015). A direct relationship between TRESK channels and the standing outward current evoked by LPA has been shown in *Xenopus* oocytes that co-express TRESK channels and the LPA_2_ receptor, where an increase in basal potassium currents was observed upon LPA application (Kollert et al., 2015). The experiments showed that LPA effects were significantly reduced if the cells were preincubated with an inhibitor of phospholipase C and eliminated by using a TRESK channel where the calcineurin binding site was mutated. These results indicated that a GPCR signaling pathway facilitates LPA's effects on TRESK (Figure 2A). Additionally, experiments in DRG neurons showed that LPA elicits background standing outward currents through TRESK-channel activation since DRG neurons from TRESK KO mice lost these currents, whereas the inward currents produced through TRPV1's activation by LPA were maintained (Kollert et al., 2015). Furthermore, DRG neurons from TRESK KO mice displayed increases in spike frequency upon LPA application, exhibiting enhanced excitability (Kollert et al., 2015). In this case, LPA has a complex effect by positively modulating TRESK channels and functioning as a pain inhibitor while activating TRPV1 that is also expressed in the same neurons producing pain. It could be hypothesized that activation of TRESK channels serves as a compensation mechanism that decreases depolarization induced through the activation of other channels (i.e., TRPV1). It is yet to be clarified under which scenario LPA can effectively produce pain by bypassing the inhibitory activity of TRESK channels. In this respect, it is known that the LPA_2_ receptor desensitizes and, since the activation of TRESK channels depends on the signaling pathway of this GPCR, it is possible that the pain inhibiting role of TRESK channels is overcome by the activation of TRPV1 by LPA finally resulting in pain.

Another K_2P_ channel with moderate expression in DRG neurons is the TREK-1 channel, which is regulated by mechanic, chemical, and thermal stimuli (Maingret et al., 1999). Similar to TRESK, it has been reported that LPA positively regulates TREK-1. The first report was obtained from COS cells transiently expressing TREK-1 where inside-out excised patches exposed to LPA exhibited TREK-1's activation, contrary to COS cells without TREK-1 expression where LPA intracellular application failed to produce currents (Chemin et al., 2005).

TREK-1 is characterized by its intrinsic voltage-dependence and its low activation at atmospheric pressure, properties that are reverted by the intracellular presence of LPA. TREK-2 and TRAAK channels are also regulated at atmospheric pressure in the presence of LPA (Chemin et al., 2005). Although, the molecular mechanism of activation of TREK-1, TREK-2, and TRAAK by LPA is still unresolved, “it has been suggested that LPA affects membrane-curvature (Figure 2C), resulting in modulation of the function of these mechanosensitive channels” (Chemin et al., 2005).

In contrast to the effect described above where intracellular LPA activates TREK-1 and which possibly depends on changes in the membrane curvature, inhibitory LPA effects on TREK-1's activation have also been reported in *Xenopus* oocytes expressing human TREK-1 channels when LPA is now extracellularly applied (Cohen et al., 2009). In this case, LPA extracellularly binds to a GPCR and triggers activation of the PLC producing the inhibition of TREK-1 (Figure 2A). When serines S315 and S348 in TREK-1 are substituted by alanines, the channels are not phosphorylated by PKC and renders them resistant to LPA's effects (Cohen et al., 2009). Although the dependence of the pain response upon inhibition of TREK-1 by LPA has not been demonstrated yet, it can be hypothesized if the channel is inhibited by LPA, a possible physiological consequence on the pain pathway would be a decrease in the threshold to pain and, under this scenario, LPA would function as an algogenic molecule through modulation of TREK-1 activity under pathological situations where LPA's extracellular levels are increased.

## Voltage-Gated Calcium Channels are Targets of LPA's Signaling Pathways

When the action potential in the pain pathway arrives to synaptic nerve terminals of primary neurons, depolarization-induced opening of Cav channels occurs (Sekiguchi et al., 2018). CaV channels are classified as high- or low-voltage-activated (HVA and LVA, respectively). They share a structural topology similar to that of Nav channels. Additionally, HVA calcium channels (Cav1.2-1.4 and Cav2.1-2.3) are composed by accessory subunits (α2δ and β), which regulate their cell surface density (Dolphin, 2016).

Several reports have shown that intrathecal LPA injection in mice produces the same effects as observed in the mice with partial sciatic nerve injury, a neuropathic pain model (Ueda, 2006). These mice display mechanical allodynia, thermal hyperalgesia, and demyelination (Inoue et al., 2004). Moreover, LPA injection and nerve injury triggers up-regulation of the α2δ1 subunit of HVA calcium channels in DRG neurons. This up-regulation can be avoided in the LPA_1_ receptor null-mice or by using a RhoA inhibitor (Inoue et al., 2004). Thus, these data demonstrate that LPA positively regulates α2δ1 expression, a subunit important for Cav2.2, which is abundantly expressed in nociceptors (N-type calcium channels; Figure 2A). Such effects are mediated through the LPA_1_ receptor, which probably enhances the excitability of sensory neurons causing neuropathic pain. Mice lacking N-type calcium-channel expression show resistance to developing neuropathic pain symptoms (Saegusa et al., 2001), in contrast to the effects of LPA that positively regulates the auxiliary subunit α2δ1 of this channel resulting in pain (Inoue et al., 2004).

Cav3.2, a LVA calcium channel is expressed in small DRG neurons (Rose et al., 2013) and it has been shown that mice intrathecally-treated with antisense oligonucleotides against Cav3.2 display antinociceptive, anti-hyperalgesic, and anti-allodynic effects (Bourinet et al., 2005). This channel is also regulated by LPA and it was demonstrated in rat DRG neurons that T-type currents following extracellular LPA application are increased through a mechanism dependent on the GPCR signaling pathway (Figure 2A) since the effect is blocked when the neurons are pre-treated with a ROCK inhibitor (Iftinca et al., 2007).

Positive regulation of Cav channels in nociceptors guarantees the release of excitatory amino acids (glutamate and aspartate) and/or neuropeptides (substance P) at their synapses with dorsal horn neurons that project to the brain (Zamponi et al., 2009); thus, Cavs play an important role in the transmission of harmful signals that may be regulated by LPA.

## Conclusion

Regulation of ion channels by endogenous molecules is key to the generation and maintenance of pain. The elucidation of the nature of these molecules and the molecular mechanisms by which they produce pain is fundamental to achieve tools to understand and control pathological processes associated to their functions. Much work is still needed to address questions on which other ion channels are regulated by this phospholipid and on how the native systems where these are expressed are effectively regulated by LPA and other substances whose levels are increased under pathological situations. For example, there is scarce information as to how this phospholipid regulates P2X3 receptors that have been shown to participate in pain (Burnstock, 2016). Only one study shows that LPA can regulate the activity of P2X3 receptors through an LPA1-mediated signaling pathway in bonce cancer pain in rats (Wu et al., 2016).

It is also important to consider that some pathophysiological conditions may produce sensitization of nociceptors and leading to a shift in the pain threshold in response to LPA (Gold and Gebhart, 2010).

## Author Contributions

RJ-C, TR, and SLM-L conceived and wrote this manuscript.

### Conflict of Interest Statement

The authors declare that the research was conducted in the absence of any commercial or financial relationships that could be construed as a potential conflict of interest.
